# PEG-POSS Star Molecules Blended in Polyurethane with Flexible Hard Segments: Morphology and Dynamics

**DOI:** 10.3390/molecules26010099

**Published:** 2020-12-28

**Authors:** Konstantinos N. Raftopoulos, Edyta Hebda, Anna Grzybowska, Panagiotis A. Klonos, Apostolos Kyritsis, Krzysztof Pielichowski

**Affiliations:** 1 Department of Chemistry and Technology of Polymers, Cracow University of Technology, ul. Warszawska 24, 31-155 Kraków, Poland; edyta.hebda@pk.edu.pl (E.H.); annagrzy95@gmail.com (A.G.); kpielich@pk.edu.pl (K.P.); 2 Department of Physics, National Technical University of Athens, Zografou Campus, 157 80 Athens , Greece; panos48al@gmail.com (P.A.K.); akyrits@central.ntua.gr (A.K.)

**Keywords:** polyurethane, POSS, glass transition, microphase separation, dielectric properties

## Abstract

A star polymer with a polyhedral oligomeric silsesquioxanne (POSS) core and poly(ethylene glycol) (PEG) vertex groups is incorporated in a polyurethane with flexible hard segments in-situ during the polymerization process. The blends are studied in terms of morphology, molecular dynamics, and charge mobility. The methods utilized for this purpose are scanning electron and atomic force microscopies (SEM, AFM), X-ray diffraction (XRD), differential scanning calorimetry (DSC), and to a larger extent dielectric relaxation spectroscopy (DRS). It is found that POSS reduces the degree of crystallinity of the hard segments. Contrary to what was observed in a similar system with POSS pendent along the main chain, soft phase calorimetric glass transition temperature drops as a result of plasticization, and homogenization of the soft phase by the star molecules. The dynamic glass transition though, remains practically unaffected, and a hypothesis is formed to resolve the discrepancy, based on the assumption of different thermal and dielectric responses of slow and fast modes of the system. A relaxation α′, slower than the bulky segmental α and common in polyurethanes, appears here too. A detailed analysis of dielectric spectra provides some evidence that this relaxation has cooperative character. An additional relaxation g, which is not commonly observed, accompanies the Maxwell Wagner Sillars interfacial polarization process, and has dynamics similar to it. POSS is found to introduce conductivity and possibly alter its mechanism. The study points out that different architectures of incorporation of POSS in polyurethane affect its physical properties by different mechanisms.

## 1. Introduction

A standard cubic siliceous core of a polyhedral oligomeric silsesquioxane (POSS, R_8_Si_8_O_6_) molecule has a typical size of approximately half a nm. Organic groups R attached on its siliceous vertices (vertex groups) form a shell which typically has a size of the same order of magnitude. The whole structure spans thus a total of 1–3 nm, and POSS moieties are often referred to as nanoparticles. Indeed, POSS have been observed to behave as conventional nanoparticles, however this is usually an effect of their self-assembly to nano-sized aggregates [1]. On the other hand, POSS can behave as relatively large complex molecules, showing for example their own glass transition [1,2,3,4]. We could therefore state that POSS lie in the grey zone between nanoparticles and large organic-inorganic hybrid molecules. This is more so, when the organic shell does not consist of relatively short organic groups, but of rather long oligomeric chains. In this case the whole structure can be described as a star polymer with a siliceous core. The most popular and commercially available star variant of POSS is the one with poly(ethylene glycol) (PEG) vertex groups of molar mass ~600 [5]. This substance has been studied in the past as additive in polymer matrices, especially with the intention to improve membrane properties [5,6,7,8,9,10,11,12]. A neutron spin echo study was performed to investigate the dynamics of the system in entangled and unentangled PEG matrices [13].

Polyurethane, on the other hand, is a family of versatile polymers synthesized on the basis of the urethane bond formed by the reaction of a hydroxyl and an isocyanate group. A standard polyurethane elastomer consists of the so-called soft and hard segments. The two types of segments at room temperature are incompatible to each other, and the standard morphology is that of hard microdomains in a soft continuous phase [14,15,16,17,18,19]. The macroscopic properties of elastomeric polyurethanes, including mechanical, thermal, electrical and dielectric ones depend strongly on the microphase separation. Note that either phase may or may not crystallize, depending on the nature, molar mass, and purity of their components. The extent of the microphase separation is strongly related to the nature of the segments, their molar mass, and—very important—their thermal history [20,21,22].

POSS moieties have been incorporated covalently in polyurethane matrices in various ways using standard urethane or urea chemistry. They have been built-in as pendent groups [23,24,25,26,27], as “beads” along the chain contour [28], or even as crosslinking moieties [29]. POSS moieties have also been blended into polyurethane systems without covalent bonding: moieties with short vertex groups can be melt blended in polyurethane [22,30,31] and PEG-POSS moieties like those utilized in this study can be present in situ during the synthesis [32]. In these works, it has become apparent that the influence of POSS on glass transition dynamics is extremely complex [33]. Please note that in this discussion we refer only to the glass transition of the soft phase. Three main mechanisms seem to be present. (i) POSS forming nm-sized aggregates can immobilize chains around them and slow down the overall dynamics, i.e., increasing the glass transition temperature *T_g_*. (ii) Glass transition of polyurethane is known to be affected significantly by the degree of microphase separation. In fully separated systems by definition the soft phase consists only of polyol segments and exhibits a similar *T_g_*. As more hard segments blend in, the soft phase is not pure anymore and, consequently, its *T_g_* increases moderately following mixing models such as that of Fox [34]. (iii) POSS are diluted in the soft phase and alter its dynamics as expected by mixing laws.

In a previous work, we studied systems where PEG-POSS moieties were blended into a model polyurethane with polytetramethylene ether glycol (PTMG) as soft phase, and hard segments consisting of the aromatic, rigid methylene diphenyl diisocyanate (MDI) and butanediol [32]. In this case POSS facilitated the development of macroscopic regions (several μm) presumably with a hard segment backbone, but in the submicron range POSS had a very minor decreasing effect on the microphase separation. The glass transition dynamics accelerated moderately, and *T_g_* dropped slightly more than expected by Fox’s equation, indicating possible development of additional free space in the soft phase. 

Traditionally, the diisocyanate contains aromatic groups, however this is not favourable for applications where biocompatibility and non-toxicity are important. Hence, interest has grown in the development of polyurethanes with aliphatic diisocyanates [35]. In past work, we have synthesized such a polyurethane based on the aliphatic and flexible hexamethylene diisocyanate, and chemically introduced in it POSS as pendent groups [35]. In this case, the effect of POSS on glass transition dynamics was quite complex. The calorimetric *T_g_* was practically unaffected, whereas the dynamic glass transition as studied by dielectric spectroscopy was slowed down. In order to resolve the discrepancy we put forward the hypothesis that the slow modes that emerged have a dielectric footprint but small to negligible thermal response, much like the immobilized polymer fraction observed in crystalline or nanocomposite systems [36,37]. The difference in this system though is that no new process emerges but there is only a disturbance of the distribution of time scales in the α process.

**Scheme 1:**
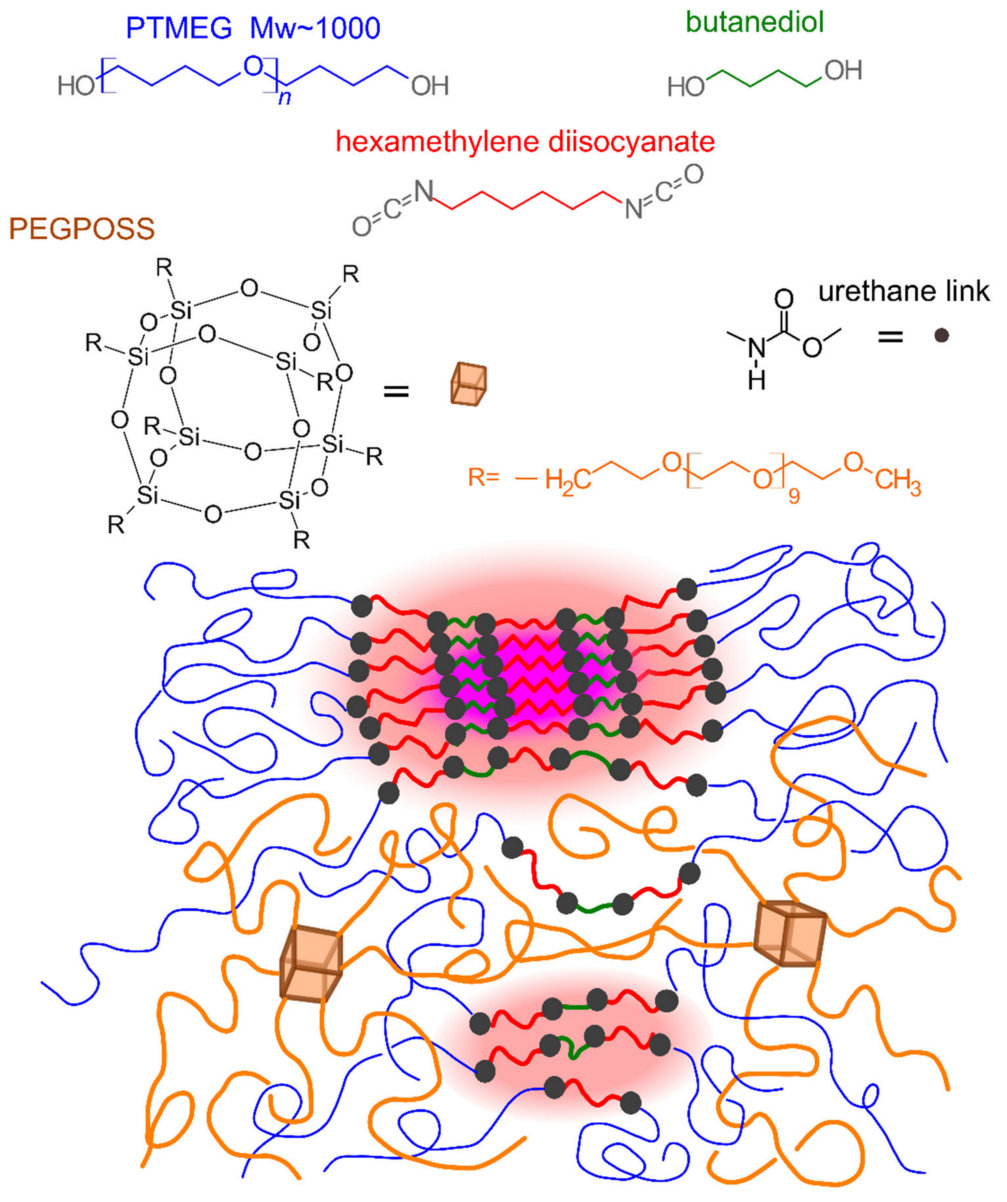
Chemical structure of the materials under investigation. Hard domains are annotated in pink background and their crystalline part in purple background. Some of the POSS vertex groups have been omitted for clarity.

In this work, we have introduced PEG-POSS star polymers, in-situ during the synthesis of the matrix with the flexible diisocyanate. The soft segments consist of polytetramethylene ether glycol (PTMG) with molar mass ~1000 g/mol. The hard segments consist of the linear hexamethylene diisocyanate (HDI) and butanediol (BD). Scheme 1 shows the chemical structure of the final systems. The mass fraction of hard segments is 50 wt%, namely their average molar mass is also ca 1000 g/mol. The materials were synthesized in a traditional two step prepolymer-chain extension procedure. POSS in fractions 0 to 10 wt% with respect to polyurethane mass was added during the prepolymerization step. Samples will be designated as PUXX, with XX being the POSS content in wt%.

We study the influence of PEG-POSS on the morphology with X-ray diffraction (XRD), scanning electron microscopy (SEM), and atomic force microscopy (AFM). Thermal transitions, with emphasis on the glass transition and the complex order-disorder process are studied by differential scanning calorimetry (DSC). Finally, we conduct a very detailed investigation of the molecular and charge mobility by means of dielectric relaxation spectroscopy (DRS). We will show that POSS has a moderate effect on the microphase separation, as well as in the glass transition dynamics, although again a discrepancy occurs between the calorimetric and dielectric response. Charge mobility is greatly enhanced as a result of the addition of PEG-POSS in the soft phase.

## 2. Results

### 2.1. Scanning Electron Microscopy

**Figure 1:**
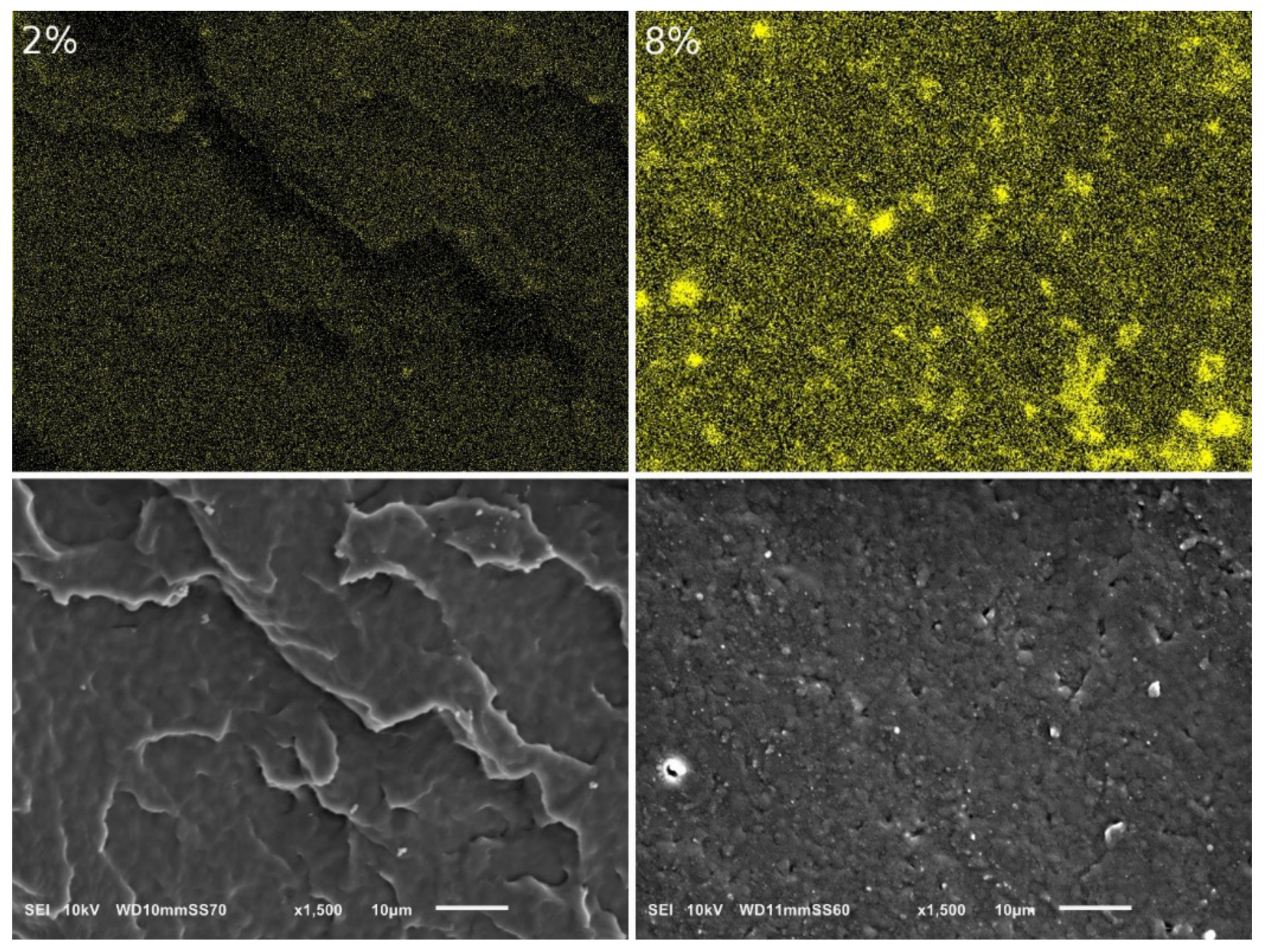
SEM images for two selected blends with 2 and 8 wt% POSS (bottom row) and corresponding energy dispersive x-ray spectroscopy (EDX) mapping at the spectral line of silicon (top row). The scale bar corresponds to 10 μm.

Scanning electron microscopy images were recorded on the surface of the elastomers, and an energy dispersive x-ray spectroscopy (EDX) mapping was performed at the spectral line of silicon, in order to study the dispersion of POSS (Figure 1).

Conventional secondary electron images for the 2 wt% blend, show scarce bright objects of size less than μm. More and bigger ones are present at a higher POSS content (8 wt%), with the largest of them having a size of 1–2 μm. EDX mapping confirms that these objects are POSS aggregates. In the case of the 8 wt% blend, except for the visible objects, EDX mapping shows more siliceous objects, presumably lying below the surface. The silicon mapping of the 2 wt% blend shows a rather homogeneous distribution of the element.

### 2.2. Atomic Force Microscopy

**Figure 2:**
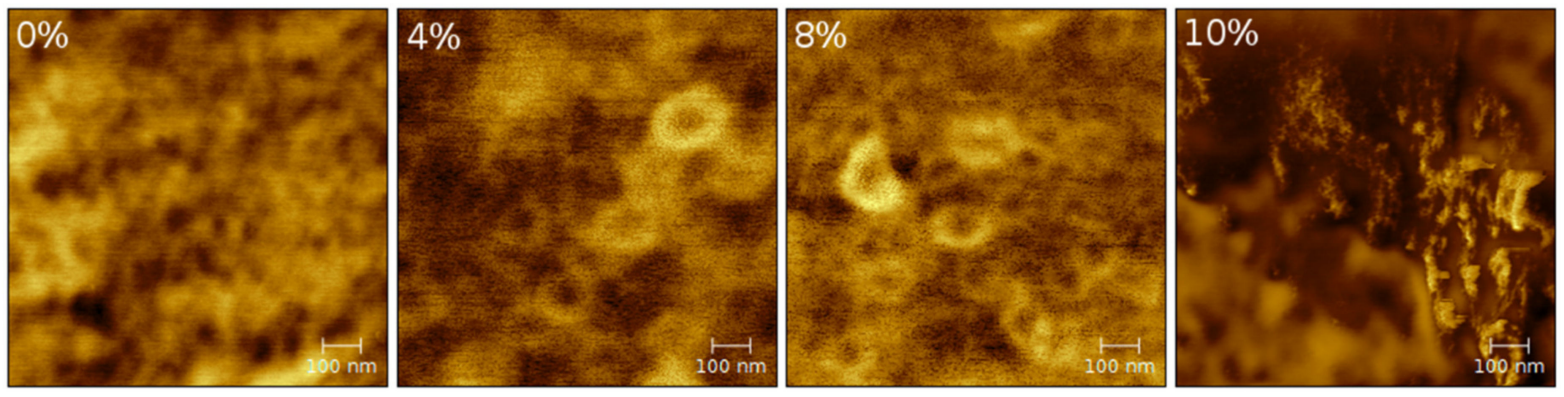
1 × 1μm AFM phase images recorded with selected blends.

Atomic force microscopy (AFM) phase images (Figure 2) shed more light into the micromorphology in the submicron length scale. Materials with 0 to 8 wt% POSS are microphase separated, however there are minor differences. The matrix shows a rather simple distribution of the two phases, whereas 4 and 8 wt% show core-shell globules, presumably corresponding to hard microdomains. The image for the 10 wt% blend does not show a microphase separated region but rather a PEG-POSS-rich region.

### 2.3. X-ray Diffraction

**Figure 3:**
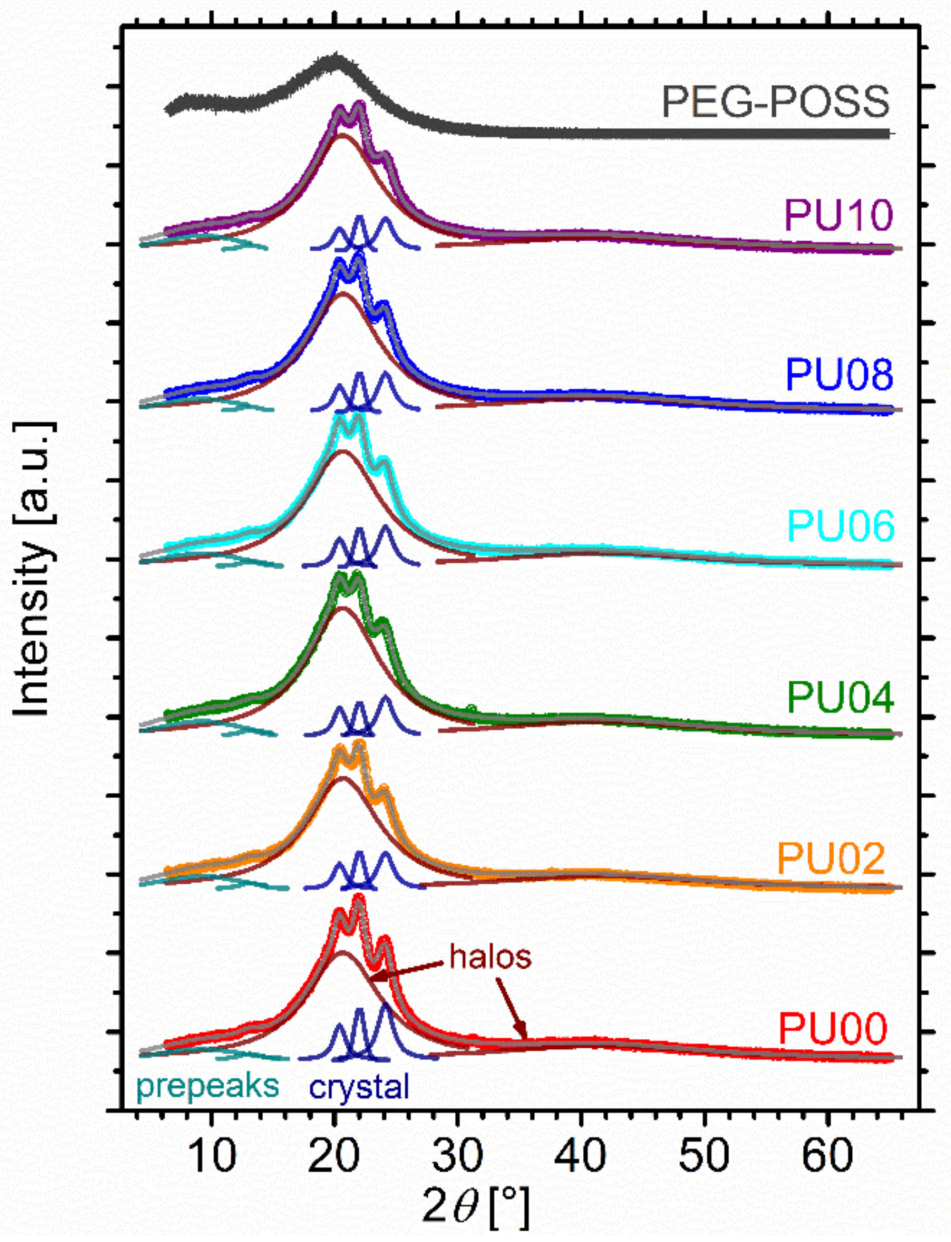
X-ray diffraction curves recorded with all materials under investigation. Gray lines are fits according to the procedure described in the text. The constituting peaks are shown incolors according to their origin. The diffractogram of PEG-POSS is added for comparison.

The diffractograms in Figure 3 resemble those observed with a similar matrix in a previous work [35].

The two main features are a complex halo including three Bragg reflections in the region 15–30° and a broad amorphous halo, centred roughly around 40°. The complex peak at small angles is attributed to average distances of atoms within the hard microdomains, whereas the halo at wider angles corresponds to the soft phase [38,39,40,41,42,43]. By the presence of Bragg peaks, we can assume that hard domains are semicrystalline. The origin of the two small peaks at angles below 15° is not clear. In this region a so-called pre-peak is known to be formed in polyurethanes and has been attributed to a reflection of urethane crystals [38] or to average distance of hard segments dissolved in the soft phase [42].

PEG-POSS is known to have a quite rich diffractogram, consisting of three amorphous halos, with the main one appearing at smaller angles (longer length scales) than those typically accessible by conventional XRD setups [32]. This reflects a multi-scale arrangement of the POSS cores at nm distances (not seen here), and arrangements of the polymeric chains, seen in the range studied here as two amorphous halos at 2*θ* ~10 and 20°. No further peaks or halos are observed at wider angles, which were not accessible by the experiment in ref [32].

Interestingly, there are no qualitative changes in the diffractograms of the blends with increasing POSS content. No new features emerged, and the existing peaks do not seem to change position. This indicates that POSS does not change radically the micromorphology of the PU matrix at nanometer level, accessible by conventional XRD. Only changes in intensity are observed. In order to quantify these changes, we fitted the diffractograms with pseudo-Voigt peaks (Figure 3). Two broad ones were used to model the halos, three sharp ones for the Bragg reflections, and another two to account for the two small peaks in the pre-peak region. In order to reduce the number of determined parameters, and improve statistical significance, during the fit we demanded that all fit parameters except for the intensities of the peaks are common for all materials.

**Figure 4:**
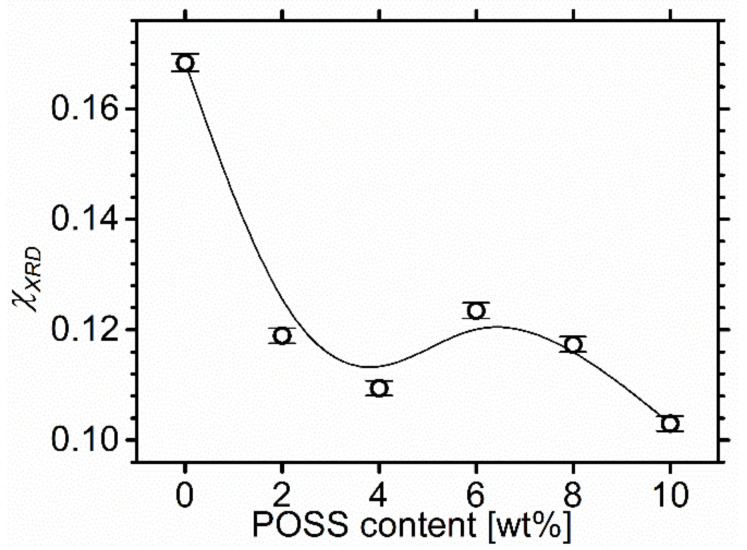
A measure of the degree of crystallinity of the hard domains, as calculated by XRD data. Details of the calculation in text. Lines are guides to the eye.

As a measure of the degree of crystallinity of the hard microdomains, we calculated the ratio:

(1) $$\chi_{XRD}\;=\;\frac{\sum A_{crystal}}{A_{HDhalo}+\sum A_{crystal}}$$

where $\sum A_{crystal}$ is the sum of the areas of the crystaline reflections, and $A_{HDhalo}$ the area of the halo related to the hard microdomains. With increasing POSS content this ratio decreases moderately indicating that PEG-POSS disturbs the development of crystallinity in the hard microdomains (Figure 4). It is not clear at this stage whether POSS penetrates the hard microdomains and disrupts their crystallinity or it merely affects the process of phase separation during curing, and thus indirectly the final morphology of the hard microdomains.

### 2.4. Differential Scanning Calorimetry

**Figure 5:**
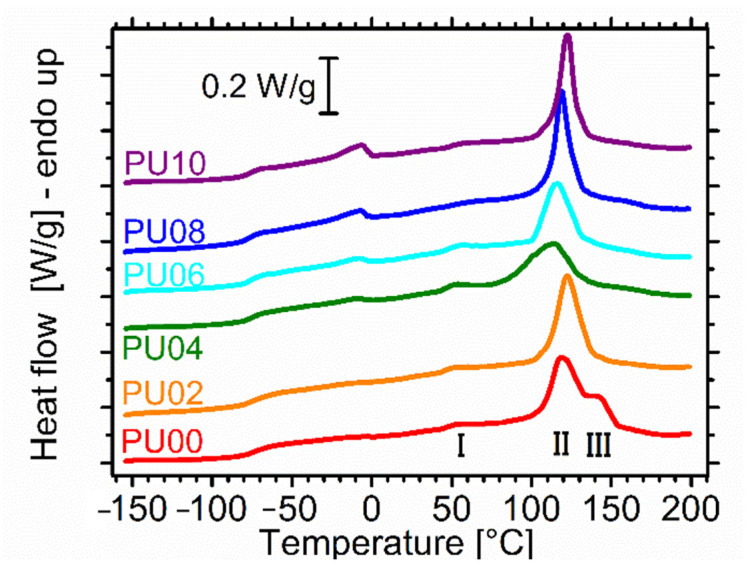
DSC heating curves for all materials under investigation. Curves are normalized to sample mass.

The differential scanning calorimetry (DSC) curves follow the paradigm of standard segmental polyurethanes (Figure 5).

Starting from low temperatures, the step-like event around −70 °C corresponds to the glass transition of the soft phase. Around −10 °C, an endothermic peak corresponds to the melting of PEG-POSS [32]. In the region 50–150 °C a series of complex endotherms corresponds to the gradual melting of hard domains and the order to disorder transition, i.e., the dissolution of hard domains into the soft phase [22,38]. 

**Figure 6:**
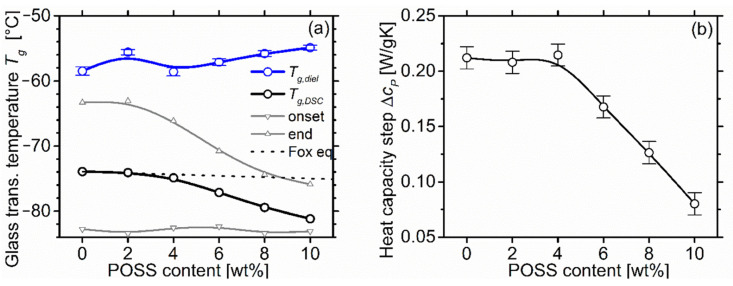
(**a**) Glass transition temperature as measured by two techniques (Dielectric Relaxation Spectroscopy, DRS, to be discussed in Section 2.5). The onset and endpoints of the glass transition step in DSC are plotted as grey lines. The dotted line corresponds to the estimation of *T_g_* according to Fox equation, taking into account that *T_g_* of POSS is −84 °C [32]. (**b**) Heat capacity change at midpoint as a function of POSS content. Lines are guides to the eye.

The soft phase glass transition temperature *T_g_* drops significantly with increasing POSS content starting at 4 wt% (Figure 6a). The drop cannot be explained fully by the plasticization of the soft phase by the POSS molecules, since the decrease is much more intense than that expected by Fox equation [34]. Interestingly, the temperature width of the step decreases considerably too, but only from the high temperature side, as the step onset stays practically unaffected. Moreover, the heat capacity step decreases significantly (Figure 6b), despite the presence of the star molecules in the soft phase fraction. Therefore, we should assume that PEG-POSS constrains the mobility of the soft phase, and interestingly the effect is more notable in the slow (high temperature) modes.

An endothermic peak emerges with increasing POSS content in the region −10–0 °C, i.e., near the melting point of pure PEG-POSS [32] (Figure 5). Hence, we should attribute it to the melting of POSS. This is in agreement with SEM and AFM results (Section 2.1, Section 2.2) which revealed POSS-rich regions. Apparently POSS can form its own domains within the current matrix, which was not the case in composites based on a matrix with an aromatic diisocyanate. The melting temperature increases weakly with the POSS content, indicating that crystallites become larger and more stable.

We now turn our attention to the thermal events above room temperature, namely effects associated with hard microdomains. We will follow the nomenclature introduced by Koberstein et al. [20,22]. The so-called peak I appears usually as a broad endothermic event, either peak or step, at temperatures 30–50 K above the annealing temperature, hence it is often named *annealing peak*. It is associated with a relaxation of the hard microdomains or with the enthalpy relaxation accompanying their glass transition [44,45]. Here it appears around 50 °C, more like a glass transition step rather than a broad step. The so called peaks II and III overlap with each other in the range 100–150 °C. Interestingly, Peak III shows a significant contribution only for the unmodified matrix, while in the blends it is hardly seen as a weak knee on the high temperature side of Peak II. We will comment further on the matter at the discussion section.

**Figure 7:**
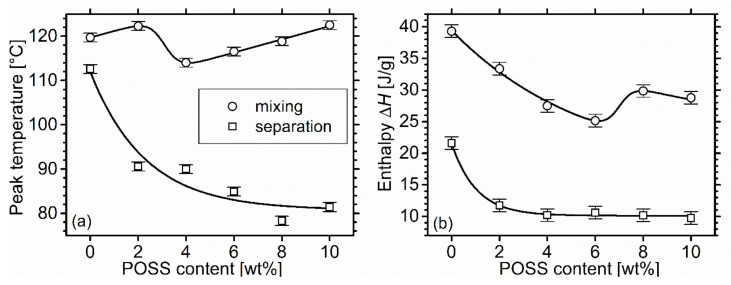
(**a**) Peak temperature and (**b**) enthalpy of microphase mixing and separation, during cooling and heating, respectively. Lines are guides to the eye.

PEG-POSS does not affect considerably the peak temperature of mixing, indicating that the size of hard microdomains is unaffected (Figure 7a). Enthalpy on the other hand (Figure 7b) decreases considerably with POSS content. Even with 1 wt% reduction of hard segment content, enthalpy of mixing is reduced by 50%. This would in general be interpreted as a decrease in the degree of microphase separation. This could be explained by the inhomogeneity in the reaction mixture and the complex entanglements introduced by the star polymer, which would severely alter the phase separation kinetics. The changes in the thermodynamics of the system due to the introduction of a third component, which is hydrophilic, should also be considered. In any case, a more detailed investigation is needed to reveal the mechanisms by which the star polymers affect microphase separation. The saturation of the values above 4 wt% is explained by the development of PEG-POSS domains.

**Figure 8:**
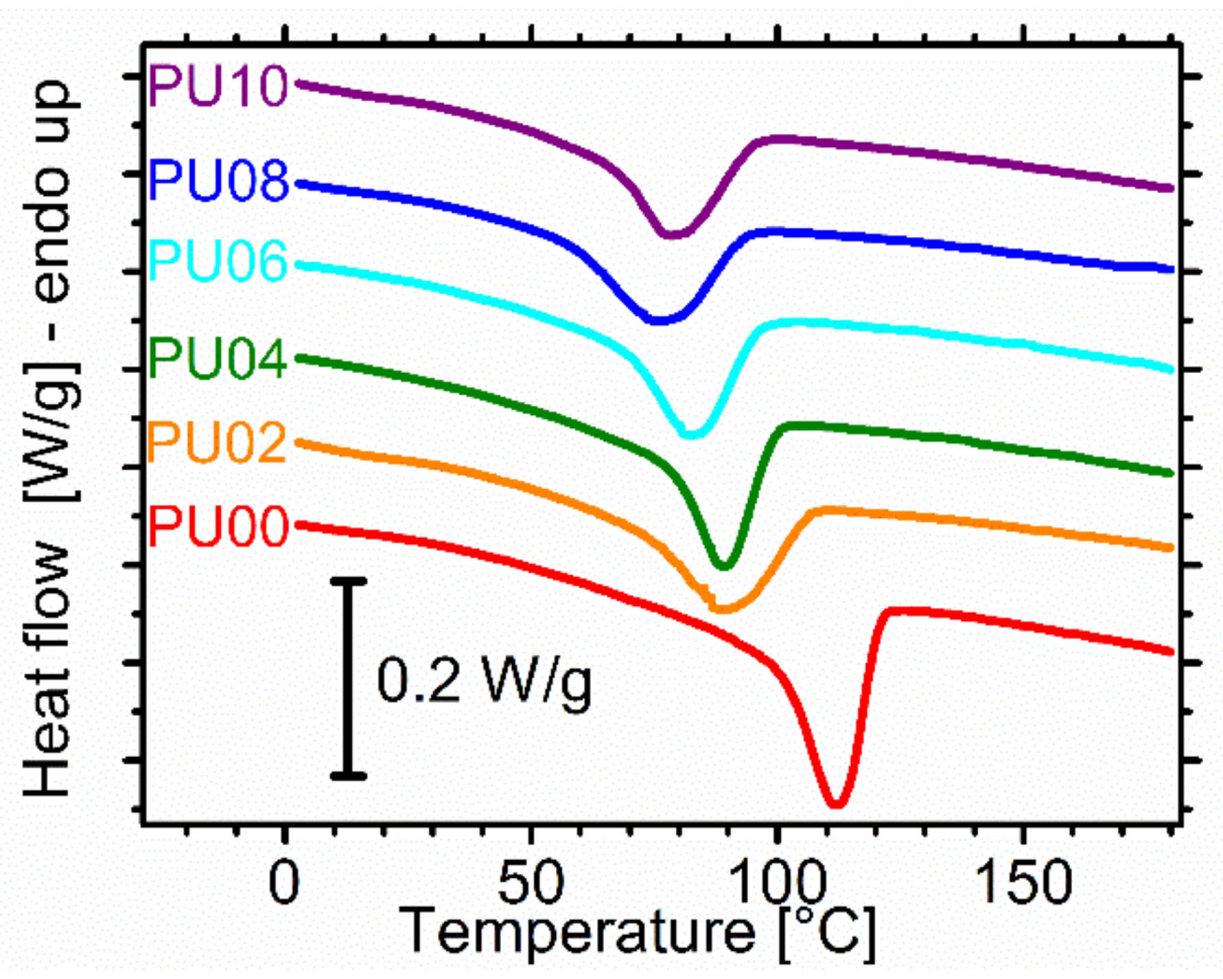
DSC cooling curves recorded with all materials under investigation. The curves have been translated for clarity.

During the subsequent cooling DSC step, an exothermic peak corresponds to the re-development of microphase separation (Figure 8). With increasing PEG-POSS content the supercooling needed for the microphase separation process increases considerably, even at 2 wt% content, while the enthalpy of microphase separation also diminishes (Figure 7, Figure 8). Interestingly, enthalpy stabilizes already after 2 wt%. A similar saturation is observed for the peak temperature, but the phenomenon is less pronounced. This behaviour is explained by the aggregation of POSS in clusters, and hence their non-availability in the bulk of the material.

### 2.5. Dielectric Relaxation Spectroscopy

Dielectric spectroscopy experiments were conducted for the study the effect of PEG-POSS on the molecular and charge mobility of polyurethane.

#### 2.5.1. Overall Behaviour

**Figure 9:**
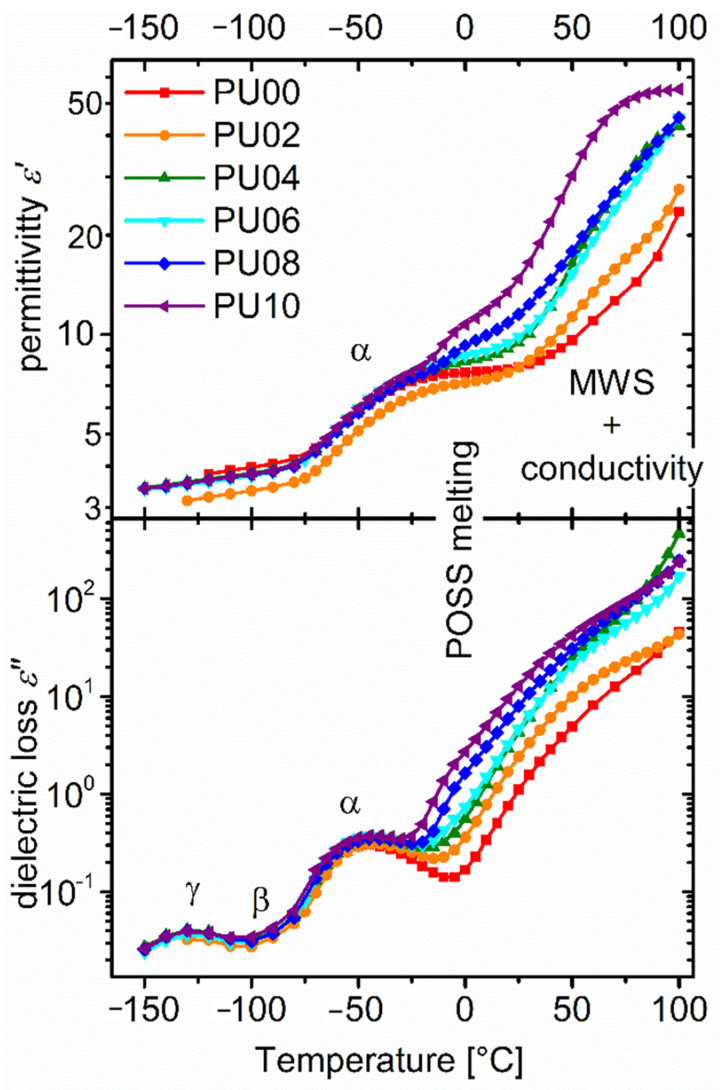
Isochronal plots of the dielectric function for the matrix and the blends, at a frequency of 10 Hz. The observed processes are annotated.

In order to illustrate the various processes probed by dielectric spectroscopy in the broad temperature range studied, we replotted the dielectric function data recorded isothermally at 10 Hz as a function of temperature, making the so-called isochronal plot (Figure 9). 

Up to ca −30 °C, we observe molecular mobility mechanisms, namely the secondary γ, and β and α relaxations. The faster γ relaxation observed as a peak in *ε″* curves is known to originate from the crankshaft motion of methylene sequences on the polyether chain [46,47], and here one could also expect contributions from the short methylene sequence on the diisocyanate. A knee on the *ε*″ curves between γ and α relaxations corresponds to relaxation β which in this case is related to the rotating motion of carbonyl on the urethane group, probed by attached water molecules [26]. The main α relaxation of the polymer, corresponding to the dynamic glass transition is seen as a prominent broad peak in the *ε″* spectra, and as a step in the *ε′* ones. None of these relaxations show prominent changes with POSS content at this scaling, although a more detailed analysis in the following will reveal some changes in the α relaxation. A complete study of the γ and β relaxations is beyond the scope of this article.

Above −30 °C, the curves of Figure 9 include phenomena related to larger length scales. A step observed in *ε′* around −20 °C for the higher loading materials, should be associated to the melting of POSS [32] and participation of the just-melted chains to molecular mobility. Steps starting in the range 20 to 50 °C, correspond to the Maxwell-Wagner-Sillars interfacial relaxation, reflecting dipoles created by the entrapment of charge carriers at interfaces between areas of different conductivity, in this case at the interfaces between hard microdomains and soft phase [48,49]. These phenomena manifest themselves as knees on the *ε″* curves, which are rather dominated by the emerging dc conductivity contribution. The charge carrier mobility phenomena seem to become much more intense with increasing POSS content. This may arise from an increased conductivity, or from the more phase separated morphology in blends with higher POSS content.

In the following we will study in detail the α relaxation as well as the phenomena related to charge transport, namely the MWS relaxation and dc conductivity.

#### 2.5.2. Dynamic Glass Transition—α Relaxation

**Figure 10:**
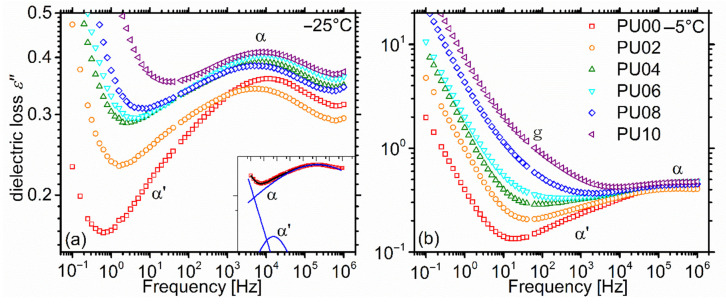
Dielectric loss *ε*″ spectra recorded with all materials at (**a**) −25 °C, and (**b**) −5 °C. Lines are fits according to the procedure described in text. The constituting functions for the spectrum of the matrix at −25 °C are shown in the inset of panel (**a**).

α relaxation is accompanied by a slower contribution α′ on its low frequency side (Figure 10a). Such a relaxation has been observed in the past in polyurethane elastomers based on various diisocyanates and polyols, and has been associated with decelerated dynamics of polyol segments anchored on hard structures [28] or to local dynamics within hard domains [50]. A steep decrease of *ε″* at low frequencies is associated with MWS and conductivity contributions, which become more intense with increasing POSS content. In fact, at the highest POSS content α′ is practically masked. At somewhat higher temperatures, a contribution emerges between the α′/α peak and the conductivity effects. We will refer to it as ‘*g*’ relaxation. At this point its origin is not clear. We will see that it is present already in the unfilled matrix, so it cannot be attributed solely to POSS. We will discuss the origin in the discussion section.

In order to quantify the relaxations in terms of timescale, strength, and width of distribution of relaxation times, we performed a fitting procedure in the α and α′ region with one Cole-Cole model function [51] for each one of them. Models describing dielectric relaxations behave as power laws at the high and low frequency limit. Hence, a power law was added to account for the contribution from free charge carrier phenomena and *g* relaxation at low frequencies [52].

Cole-Cole model is described by the equation:

(2) $$\varepsilon^{*}\left(f\right)\;=\;\varepsilon_{\infty }+\frac{\Delta \varepsilon }{1+{\left(i\frac{f}{f_{0}}\right)}^{a}}$$

In this equation, Δ*ε* is the strength of the relaxation, and *f_0_* its characteristic frequency. The exponent *a* is related to the width of the relaxation in the frequency domain. *a* = 1 corresponds to the single relaxation time Debye model and and lower values to broader distributions of relaxation times. In order to reduce the number of evaluated parameters and improve statistical significance, for each sample we imposed the constraint that the exponent of the power law and the exponent *a* for α′ remain constant throughout the fitted temperature region.

**Figure 11:**
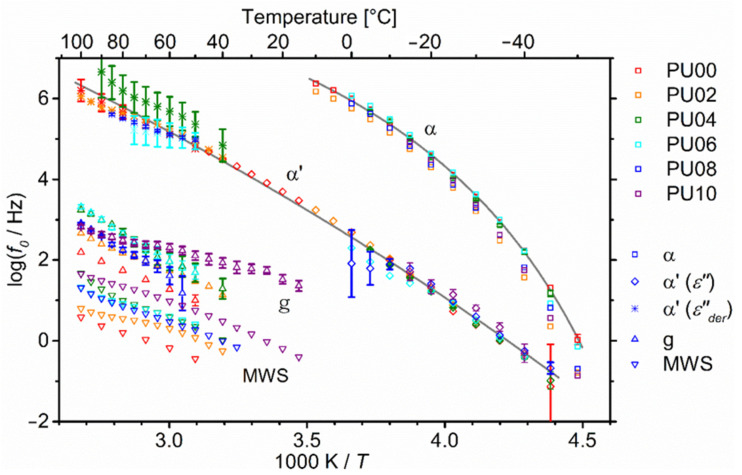
Arrhenius plot (activation diagram) for all the procedures identified in the dielectric spectra. Lines are fits of Vogel Fulcher Tammann equations (details in text). Error bars are shown only if larger than the corresponding point.

As expected, the traces of α relaxation on the activation diagram (Figure 11) follow a concave behaviour, characteristic of cooperative dynamics. The dependence on POSS content is very weak. In order to quantify the effects, the Vogel Fulcher Tammann (VFT) equation was fitted to the data, in a form proposed by Angel [53,54]:

(3) $$f_{0}\;=\;f_{\infty }\cdot e^{-\frac{DT_{0}}{T-T_{0}}}$$

In this equation, $f_{\infty }$ is the high temperature limit of the characteristic frequency also known as phonon frequency, $T_{0}$ the so-called Vogel Temperature at which the relaxation time becomes infinite, and D the so called strength parameter related to the curvature of the VFT trace. The dependence of parameters $f_{\infty }$ and D on POSS content is within the accuracy of the fit (Table 1). Despite the rather large errors it can be said that *T_0_* increases weakly with POSS content.

In order to compare with the results by calorimetry, we calculated a measure of the glass transition temperature, i.e., the so-called dielectric *T_g_* by extrapolating the traces according to VFT equation to the frequency corresponding to a relaxation time of 100 s ($f_{g}=\frac{1}{2\pi \cdot 100s}$). The results are shown in Figure 6a. Contrary to the clear decreasing trend of the calorimetric *T_g_*, the dielectric one shows a very weak but observable increasing trend. Such a discrepancy is quite rare, and we will try to resolve it in the discussion section in light of the results to follow.

**Figure 12:**
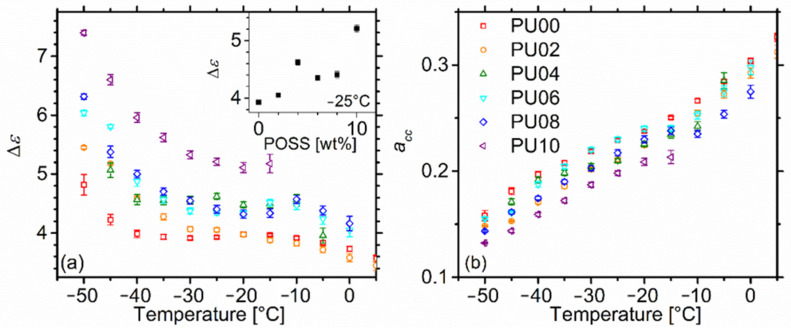
Dependence of (**a**) Δε and (**b**) shape exponent a of α relaxation as a function of temperature. The onset of (**a**) shows the dependence of Δε at −25 °C on POSS content.

The strength Δ*ε* decreases with temperature, as is expected for the segmental relaxation that is related to glass transition (Figure 12a). This is an effect of thermal motions inhibiting the polarizability of the material. With increasing POSS content, despite some scattering, an increasing trend of the order of 25% in the studied range is observed with POSS content (inset). Meanwhile, α relaxation is extremely broad at low temperatures with an exponent *a* around 0.15 indicative of a very heterogeneous nanomorphology. The soft phase of polyurethanes (the dynamic glass transition of which gives rise to this α relaxation) consists of polyol segments and diluted hard segments with various lengths. In that respect, the inhomogeneity, even in the nanoscale, is enormous. With increasing temperature, this inhomogeneity decreases. With increasing POSS content the exponent *a* shows a weak decreasing trend indicative of slightly more inhomogeneous systems [55].

#### 2.5.3. Higher Temperature Relaxations—Maxwell-Wagner-Sillars Process

At temperatures above 0 °C, charge carrier effects dominate the spectra (Figure 9), namely the dc-conductivity and the MWS relaxation. We remind also that a broad *g* relaxation faster than α but slower than MWS was observed at relatively low temperatures (Figure 10), and so far has not been studied.

**Figure 13:**
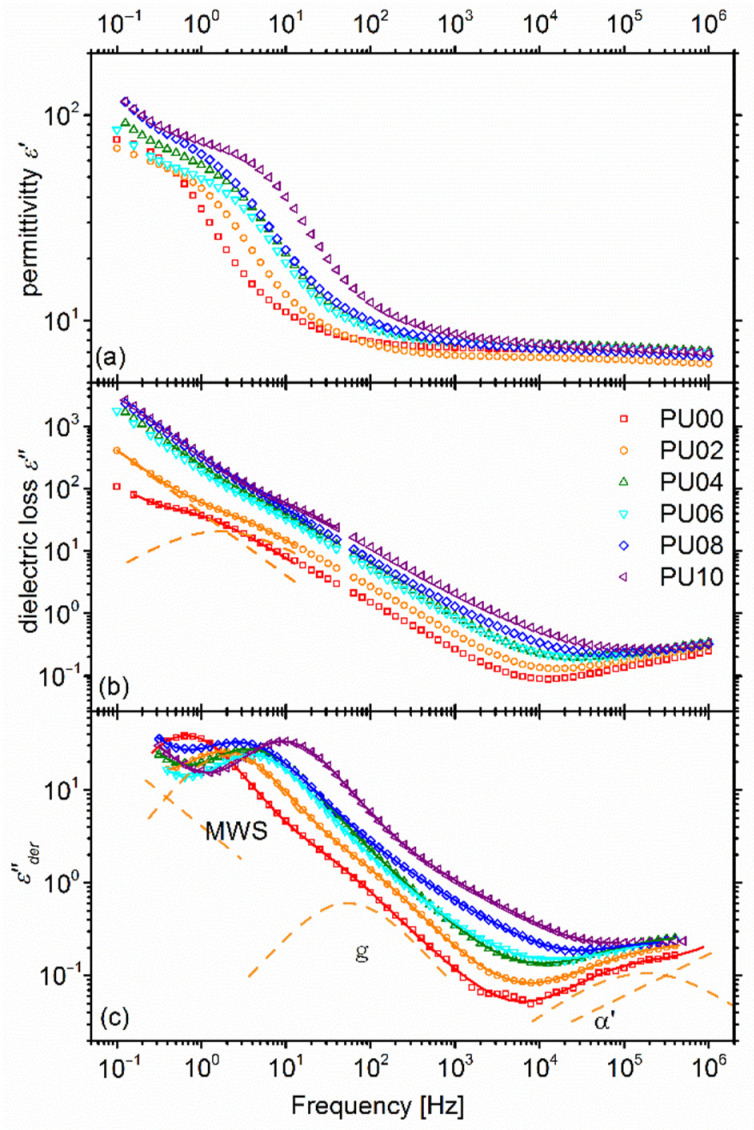
(**a**) Real and (**b**) imaginary parts of the dielectric function at 60 °C for all materials under investigation. (**c**) $\varepsilon_{der}^{\prime \prime }\;$ spectra as calculated according to the method described in text. Cntinuous lines are fits according to the procedure described in text. Dashed lines are individual components of the fits for the sample with 2 wt% POSS.

At 60 °C, dc conductivity dominates the *ε*″ spectra, with a contribution of the form $\varepsilon^{\prime \prime }\propto f^{-1}$ (Figure 13b). Clearly the conductivity increases with POSS content, but we will return to that upon a formal analysis. A knee on the conductivity contribution corresponds to the MWS relaxation. The *g* relaxation is completely masked by conductivity. At the high frequency end of the window, an upturn corresponds to the α′ relaxation superimposed on a slope due to the low frequency tail of the α relaxation.

In order to facilitate the analysis, we relied to a methodology put forward by Van Turnhout and Wübbenhorst [56]. DC-conductivity does not affect *ε*’ spectra. Indeed, they form well defined steps in the region of MWS relaxation (Figure 13a). A small upturn at low frequencies marks the onset of stronger effects related to charge carriers, most likely the parasitic electrode polarization.

Now, according to the methodology in ref. [56], based on Kramers-Kronig relationships, we can calculate an approximation of the “conductivity free” *ε″* as:

(4) $$\varepsilon_{der}^{\prime \prime }=\;-\frac{\pi }{2}\frac{\partial \varepsilon^{\prime }\left(\omega \right)}{\partial \ln\omega }$$

In addition to being conductivity free this approximation produces somewhat narrower peaks, provides slightly better resolving power to our analysis.

Indeed, the $\varepsilon_{der}^{\prime \prime }$ spectra show a strong and narrow MWS peak with a contribution appearing as a weak shoulder on its high frequency slope, which corresponds to the g relaxation (Figure 13c). A fitting was performed with a sum of three Cole-Cole model functions (Equation 2), modified to correspond to the $\varepsilon_{der}^{\prime \prime }$ formalism, along with two power laws to account for phenomena at frequencies below and above the experimental window (Figure 13c).

In order to reduce the optimized parameters and provide statistically significant results, we imposed the constraints that the strength Δ*ε* and width exponent *a* of the α′ relaxation, and the exponents of the power laws remain constant throughout the temperature window, in which both MWS and g relaxations are visible.

The results on the time scale of relaxations are included in the Arrhenius plot of Figure 11. Despite some scattering, the points for α′ relaxation lie on the extrapolation of the low temperature points, and there is no indication that POSS affects its dynamics. Looking at the overall trace, it is very likely that it is concave, however with very small curvature, indicating a mildly cooperative character. A common fit of the VFT equation in the form proposed by Angel (Equation (3)), returns the values $\log\left(\frac{f_{0}}{Hz}\right)\;=\;13.6\pm 0.6$, D = 63 ± 16, and T_0_ = 78 ± 11 K.

Both MWS and *g* relaxation accelerate with increasing POSS content, and demonstrate similar temperature behaviour, i.e., similar slopes in the activation diagram (Figure 11). The MWS trace shows a small change of slope around 70–80 °C, possibly related to an early slow onset of the order to disorder transition observed by DSC to start around 100 °C (Figure 5).

**Figure 14:**
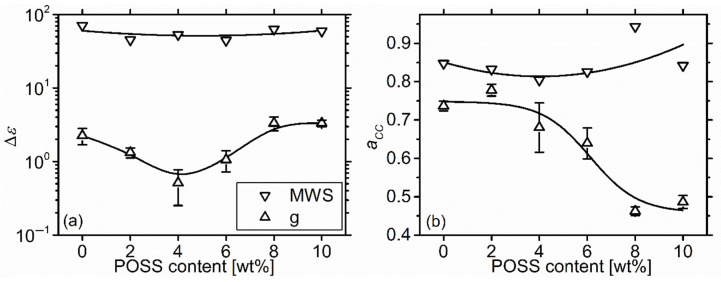
Strength Δε and shape exponent a of the MWS and g relaxation as a function of POSS content at 60 °C. Lines are guides to the eye.

Discussing in detail the dependence of Cole-Cole parameters on temperature is beyond the scope of this article. Nevertheless, we would like to show their dependence on POSS content at the representative temperature of 60 °C (Figure 14). Neither Δ*ε* nor *a* of MWS relaxation shows not show any strong trends with POSS content. However, these parameters for g relaxation seem to depend quite strongly on POSS content. Δ*ε* shows a minimum at 4 wt%, while around the same value the relaxation becomes much wider with *a* dropping from around 0.75 to 0.5 in a roughly stepwise way. Thus, POSS seems to have an effect on this relaxation, however its exact nature is still unclear. The possibility that more than one processes contribute to g relaxation should also not be excluded (see discussion section).

#### 2.5.4. Dc Conductivity

**Figure 15:**
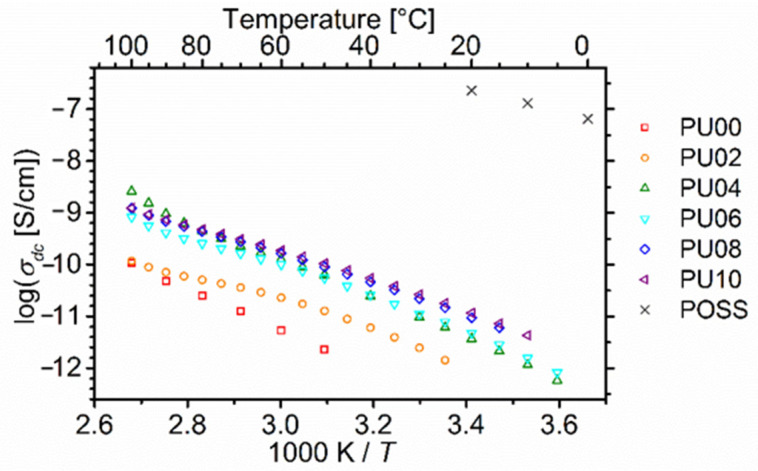
Arrhenius plot (activation map) of dc conductivity for all materials under investigation. A few points for PEG-POSS have been included for comparison. Data for POSS are based on re-analysis of data published in [32].

In order to calculate dc conductivity $\sigma_{dc}$, a fitting procedure was performed on the *ε″* data, in the low frequency region, but for frequencies higher than the onset of electrode polarization in *ε′* spectra. A term of the form:

(5) $$\varepsilon^{\prime \prime }\left(f\right)\;=\;\frac{\sigma_{dc}}{\varepsilon_{0}\cdot 2\pi f}$$

was fitted to the data, along with a Cole-Cole term (Equation (2)) to account for the MWS relaxation. In Equation (5)), $\varepsilon_{0}=8.85\cdot 10^{-12}F/m$ is the permittivity of free space. The parameters of Cole-Cole term where fixed to the values obtained by the fits of the $\varepsilon_{der}^{\prime \prime }$ spectra. The results for $\sigma_{dc}$ are included in the Arrhenius map of Figure 15. A similar procedure was followed for dielectric data recorded with neat PEG-POSS in a previous work [32], in order to provide a comparison. Note that POSS has $\sigma_{dc}$ more than five orders of magnitude higher than the neat polyurethane.

The traces of dc conductivity of the polyurethanes on the Arrhenius plot have a non-standard shape, presumably due to the thermal event at ~50 °C, which is identified as the peak I of microphase mixing procedure [20,44,57] and associated to the relaxation of hard microdomains (Figure 15).

At temperatures below this point addition of the much more conductive POSS increases remarkably the dc conductivity. The trend saturates at high POSS contents, presumably due to the incomplete mixing of POSS in the PU matrix. With the exception of the matrix where we don’t have enough data points, the traces show a concave behaviour, characteristic of cooperative processes. In order to quantify this, we fitted a VFT-like equation:

(6) $$\sigma_{dc}\;=\sigma_{\infty }\cdot e^{-\frac{DT_{0}}{T-T_{0}}}$$

where $\sigma_{\infty }$ is the high temperature limit of dc conductivity, D a strength parameter, and $T_{0}$ a temperature where charge mobility ceases. The results are shown in Table 1 and interestingly they do not show a monotonic behaviour. Contrary to the actual conductivity below 60 °C, $\sigma_{\infty }$ initially increases, and then drops at high POSS contents, and this might reflect that inhomogeneity due to POSS-rich regions inhibit charge mobility. At the same time, the strength parameter increases (along with its inaccuracy) and then decreases again, indicating that the conduction mechanism in the intermediate loading blends are less cooperative (more Arrhenius-like) than the others. This might be related to a change in the conduction mechanism, from a cooperative mechanism controlled by segmental mobility, to a thermally activated in a homogeneous medium, to a cooperative again in a material with POSS-rich regions. $T_{0}$ shows a clear decreasing trend, contrary to what was observed for the Vogel Temperature of the dynamic glass transition, which shows only minor changes (Table 1). However, this can be attributed to the morphological changes in the soft phase imposed by the melting of POSS-rich regions, which takes places between the temperature ranges where α relaxation and dc-conductivity are observable.

Above 55 °C, conductivity shows a slight downwards deviation from its initial VFT behaviour, possibly because hard segments dilute in the soft phase and inhibit the overall charge mobility. Nevertheless, the overall trend is increasing with POSS content with the exception of the 4 wt% blend which exhibits particularly high values. This might also be related to the change of conduction mechanism which was hypothesized to take place at intermediate POSS loadings.

## 3. Discussion

The morphology of the polyurethane systems at hand is in accordance to that of typical segmented polyurethanes, with hard microdomains distributed in a soft phase [19,20,26,29,35,58,59]. The thermal footprint of the order-disorder transition of the dissolution of hard microdomains into the soft phase is known to consist of three exothermic peaks. Following the notation of the seminal works of Koberstein’s group: Peak I is a broad and weak one at temperatures 30–40 K above the annealing temperature, and has been also associated to enthalpy relaxation of hard microdomains, accompanying its glass transition [20,44,57]. Peaks II and III are stronger and narrower exotherms occurring at higher temperatures. Peak II is associated with the dissolution of amorphous hard microdomains [38]. Peak III at slightly higher temperatures has been associated with melting and immediate dissolution of crystalline hard microdomains while a recrystallization process should not be excluded [38,57]. Interestingly in the case at hand, peak III is only visible clearly for the matrix but for the blends it is hardly seen as a shoulder on the high temperature side of peak II (Figure 5). Meanwhile the degree of crystallinity of the hard domains (Figure 4) as estimated by XRD is indeed much lower in the blends, providing further experimental evidence to the aforementioned assignment of exotherms. Furthermore, peak I in our case also resembles a weak step providing further support for its connection to the glass transition of the hard microdomains. In agreement to that, Fernandez-d’Arlas and Eceiza have observed a similar peak at the same position in HDI-butanediol homopolymer [60]. This transition has also some effect in charge mobility (Figure 11, Figure 15) indicating also a relationship with morphological changes.

POSS seems to disrupt the development of crystallinity in the hard microdomains (Figure 4). It is unlikely that POSS actually penetrates the hard microdomains as no radical changes are observed in their glass transition temperature (peak I) or dissolution temperature (peak II) (Figure 5). So, the reason for the decrease of degree of crystallinity should be sought in more detail by morphological techniques such as SAXS in future work.

The dynamic glass transition (α relaxation) of the system at hand is accompanied by a slower contribution α′, as has been observed in several polyurethane systems with PTMG as soft segment and various types of hard segments [21,25,26,29,50,61] as well as in semicrystalline PTMG [21]. The origin of this relaxation is not clear and has been attributed to chains anchored on immobile structures (hard microdomains, nanoparticles, crystallites) [21,28] or to local dynamics within hard domains [50]. By expanding the temperature region where this relaxation can be detected, using a more sophisticated analysis we provide evidence that this relaxation is likely cooperative, supporting its attribution to glass transition-like dynamics.

Addition of PEG-POSS seems to reduce the calorimetric *T_g_* by a few degrees, resembling what has been observed in a similar system with rigid hard microdomains [32]. Interestingly, the dynamic glass transition in the current case is found practically unaffected in terms of time scale. This rare discrepancy has been observed in PLLA nanocomposite systems [62,63]. It was also observed in a system of the same matrix as in the current study, but with POSS tethered along the polyurethane chain as chain extender. In this latter case however it was the dielectric *T_g_* that increased with the calorimetric one being relatively constant [35]. Meanwhile, it was shown that α relaxation turned significantly asymmetric with increasing POSS content, while migrating to lower frequencies. The observations were attributed to the emergence of slow modes with strong dielectric, but weak thermal response. A similar assumption can be made here. DSC analysis showed that while *T_g_* increased with addition of POSS, the onset of the step remained practically stable, and the end decreased by quite a lot, accompanied by a significant drop of the $\Delta c_{P}$ (Figure 6). This indicates that POSS suppresses the thermal response of the slow components (components with long relaxation times), but their dielectric response seems to remain unaffected, if not enhanced.

A new relaxation referred to as *g*, accompanies the Maxwell Wagner Sillars (MWS) relaxation, being faster, broader, and by one order of magnitude weaker. The fact that g and MWS have similar dynamics in terms of activation energy points to the hypothesis that g is also related to charge mobility. Such a double MWS relaxation has been observed in a system with multiple interfaces [64]. In the case at hand a second type of interfaces emerges with increasing POSS content between polymer and POSS-rich domains. At lower contents a possible origin is the interfaces between crystalline and amorphous hard domains, or between crystalline hard domains and the soft phase. This assignment is supported by the low intensity of g relaxation at intermediate contents and its different apparent activation energy at the highest POSS content. Hence, a hypothesis that in fact g possibly includes two contributions with similar timescales can be formed, but of course requires further experimental evidence.

## 4. Materials and Methods

### 4.1. Materials

Hexamethylene diisocyanate (HDI, purity >99%) was purchased from Sigma-Aldrich (Poznań, Poland) and used as received. Poly(tetramethylene glycol) (PTMG) with molar mass 1000 g/mol was acquired from Invista (Warsaw, Poland, commercial name Terathane^®^), 1,4-butanediol and Sn(Oct)_2_ as catalyst was obtained from Sigma-Aldrich and PEG-POSS was purchased from Hybrid Plastics Ltd. (Hattiesburg, MS, USA, cat. no. PG1190, cage content >92%). Polyether diol, and PEG-POSS were dried under vacuum at 80 °C, chain extender was dried under vacuum at 40 °C for 24 h before the reaction.

### 4.2. Synthesis

The two-step prepolymer method was utilized in a fashion similar to this described in ref. [35]. In the first step, PTMG and PEG-POSS were added in a three-necked round bottom flask and heated to 80 °C. They were mixed until the solution was clear. Then HDI was added dropwise, followed by Sn(Oct)_2_ catalyst. The reaction continued in Ar atmosphere, and its progress was monitored by ATR-FTIR every 15 min, until the NCO band in the spectra was stabilized. The amount of unreacted NCO groups was determined by dibutylamine back titration, and an appropriate amount of chain extender (butanediol) was then added in the reaction mixture. The reaction was allowed to proceed under stirring for 2 min, and the mixture was poured on pre-heated steel moulds, and placed in a vacuum oven. After 30 min of degassing, the temperature was increased to 100 °C and allowed to cure for 24 h. Materials with 0 to 10 wt% PEG-POSS were prepared. Mass fraction of hard segments with respect to polyurethane component was always 50 wt%. Batches of ~30 g were prepared.

### 4.3. Scanning Electron Microscopy

Scanning electron microscopy images were recorded with a JSM-6010LA microscope (Jeol, Warsaw, Poland) equipped with an energy dispersive X-ray analysis (EDS) detector. Images where taken on cryofractured samples, coated with gold. Secondary electron images at 10 kV, as well as EDS mappings on the Si spectral line were taken to study the distribution of POSS particles in the matrix.

### 4.4. Atomic Force Microscopy

Atomic force microscopy images were recorded on selected samples on the surfaces produced by the blade of a microtome. A Veeco (currently Bruker, Poznań, Poland) Innova microscope was used in tapping mode. A tip with resonance frequency ~300 kHz was used. Scans were performed on 1 × 1 μm canvas with resolution 512 × 512 pixels. Scanning frequency was 1 line per second.

### 4.5. X-ray Diffraction

Diffractograms were recorded with a Bruker D2 phaser diffractometer, on the surfaces of the as produced polyurethane sheets.

### 4.6. Differential Scanning Calorimetry

Differential scanning calorimetry curves were recorded with a Q20 calorimeter (TA, Newcastle, DE, USA) purged with He and calibrated with Indium and sapphire standards. Samples were cooled down to −150 °C at 10 K/min and subsequently heated up to 200 °C at K/min to study thermal transitions on the as received materials. The melt was then cooled down to 0 °C at 10 K/min to observe how POSS affect the phase separation process. Specimens of 5–8 mg were used, placed in standard aluminium pans.

Glass transition temperatures *T_g_* and heat capacity steps $\Delta c_{P}$ were calculated at midpoint using the proprietary TA Universal Analysis software. Peak temperatures and enthalpies were estimated using linear baselines.

### 4.7. Dielectric Relaxation Spectroscopy

Dielectric function spectra were recorded with an Alpha Analyzer (Novocontrol, Montabaur, Germany) in the range 10^−1^–10^6^ Hz. The temperature was controlled by a Quatro cryosystem of the same manufacturer. Spectra were recorded in the range −120 to 100 °C in heating steps of 5 or 10 K. As-received specimens of thickness ~1mm were placed between polished brass electrodes of 20 mm diameter.

## 5. Conclusions

A POSS variant with oligomeric PEG vertex groups was blended in a segmental polyurethane matrix with PTMG soft phase and flexible hard segments, based on the aliphatic hexamethylene diisocyanate. No significant changes are observed in the microphase separation. The apparent glass transition temperature seems to decrease and become significantly narrower, however by elimination only of the slow (high temperature/long relaxation time) components. On the contrary, the dynamic glass transition is hardly affected in terms of time scale and it becomes rather broader. A hypothesis towards the resolution of this discrepancy is that POSS arrests the thermal response of slow modes of glass transition but enhances their dielectric one. Slow modes appear to have stronger dielectric but weaker thermal response than the fast ones. The slow modes seem to be affected more by POSS.

The star-polymers with a siliceous core affect the morphology and molecular mobility of the matrix with different mechanisms as compared to POSS pendent on the hard segments [35]. The former rather plasticize the matrix, whereas the latter impose more complicated effects, including anchoring of chains and suppressing microphase separation. This indicates the significance of chain architecture when it comes to the effects of POSS on polymer matrices. From the methodological point of view, an approach of the analysis of dielectric spectra involving the derivative of permittivity with frequency, provides better insight into molecular dynamics otherwise masked by conductivity. Thanks to it, a relaxation named α′ slower than the main α, observed in several polyurethane systems and of controversial origin, is found to exhibit a very weak cooperative character.

A relaxation referred to as g, not typically observed in such systems, accompanies the Maxwell Wagner Sillars relaxation and follows its dynamics. It is proposed that it actually involves two contributions from interfaces: (i) between amorphous and crystalline hard domains or soft phase and hard domains and (ii) between polyurethane and POSS-rich domains. Clarification of its origin would be interesting in future work.

## Figures and Tables

**Table 1:** Vogel Fulcher Tammann parameters for the α relaxation and conductivity of the blends. Conductivity parameters have been determined for temperatures below peak I.

POSS Content [wt%]	α Relaxation			dc Conductivity		
	log(*f_∞_*/Hz)	*D*	*T_0_* [K]	log(*σ_∞_/*[S/cm])	*D*	*T_0_* [K]
0	10.6 ± 0.5	5.2 ± 0.9	184 ± 4	-	-	-
2	11.1 ± 0.5	5.9 ± 0.9	184 ± 3	−7.7 ± 0.1	3.7 ± 0.4	213 ± 4
4	11.5 ± 0.6	6.7 ± 1.2	178 ± 5	−6.8 ± 0.3	5.6 ± 0.7	192 ± 5
6	10.7 ± 0.5	5.0 ± 0.7	186 ± 3	−4.6 ± 1.0	19 ± 9	134 ± 24
8	10.3 ± 0.5	4.7 ± 0.6	188 ± 3	−5.1 ± 0.3	14 ± 2	142 ± 9
10	10.5 ± 0.5	4.7 ± 0.7	189 ± 3	−5.7 ± 0.2	9.6 ± 1.3	161 ± 7

## Data Availability

Samples of all the compounds are available from the authors.

## Sample Availability

Samples of the compounds are available from the authors.
